# Palliative care provision for patients with chronic obstructive pulmonary disease

**DOI:** 10.1186/1477-7525-5-17

**Published:** 2007-04-03

**Authors:** Abebaw Mengistu Yohannes

**Affiliations:** 1 Department of Physiotherapy, Manchester Metropolitan University, Elizabeth Gaskell Campus, Hathersage Road, Manchester, M13 0JA, UK

## Abstract

Chronic obstructive pulmonary disease (COPD) is a major cause of disability, morbidity and mortality in old age. Patients with advanced stage COPD are most likely to be admitted three to four times per year with acute exacerbations of COPD (AECOPD) which are costly to manage. The adverse events of AECOPD are associated with poor quality of life, severe physical disability, loneliness, and depression and anxiety symptoms. Currently there is a lack of palliative care provision for patients with advanced stage COPD compared with cancer patients despite having poor prognosis, intolerable dyspnoea, lower levels of self efficacy, greater disability, poor quality of life and higher levels of anxiety and depression. These symptoms affect patients' quality of life and can be a source of concern for family and carers as most patients are likely to be housebound and may be in need of continuous support and care. Evidence of palliative care provision for cancer patients indicate that it improves quality of life and reduces health care costs. The reasons why COPD patients do not receive palliative care are complex. This partly may relate to prognostic accuracy of patients' survival which poses a challenge for healthcare professionals, including general practitioners for patients with advanced stage COPD, as they are less likely to engage in end-of-life care planning in contrast with terminal disease like cancer. Furthermore there is a lack of resources which constraints for the wider availability of the palliative care programmes in the health care system. Potential barriers may include unwillingness of patients to discuss advance care planning and end-of-life care with their general practitioners, lack of time, increased workload, and fear of uncertainty of the information to provide about the prognosis of the disease and also lack of appropriate tools to guide general practitioners when to refer patients for palliative care. COPD is a chronic incurable disease; those in an advanced stage of the disease pursuing intensive medical treatment may also benefit from the simultaneous holistic care approach of palliative care services, medical services and social services to improve quality of end of life care.

## Introduction

Chronic obstructive pulmonary disease (COPD) is a major cause of morbidity and mortality worldwide [1,2]. Studies in the US have reported death rate from COPD doubled in the past two decades while significant decline occurred in deaths rate from other chronic diseases, for example, chronic heart disease and stroke [3,4]. The trends of increasing annual death rate of COPD was also marked by the first time the number of women dying from COPD surpassed men [5] in contrast to demographic changes in the general population. In the same period healthcare utilization was exponentially growing because patients with severe COPD were hospitalized three to four times per year and utilization of intensive care units for patients admitted with acute exacerbations of COPD in developed countries [1,4-6]. In the US in 2005, the National Heart, Lung and Blood Institute reported that the annual direct medical costs for COPD were about $21.8 billion and indirect costs (restricted days, lost workdays, and productivity) estimated at $17 billion respectively [7]. Au and co-workers [8] examined retrospectively patients who died in the previous six-months with advanced stage COPD in comparison with lung cancer patients and their healthcare utilization. They found that patients with COPD had 'twice the odds of being admitted to an intensive care unit and received fewer opiates and benzodiazepine compared with patients with lung cancer'. The total direct medical costs were $4,000 higher for COPD patients compared with lung cancer patients because of the intensive care unit utilization [8]. A recent population survey that investigated the health status of COPD patients in five European countries and US have reported that a significant but minority of European Union patients reported that their 'health status was worse than death' because of significant impairment in physical daily activities, severe dyspnoea, fatigue, pain and psychological morbidity due to anxiety and depressive symptoms [9].

In patents with severe COPD (defined forced expiratory volume in one second, FEV_1 _< 1 litre), inpatient mortality of 7.4% and 90 day mortality of 15% [10] and one year mortality rate for elderly COPD patients range from 36% [11] to 59% reported in patients admitted to intensive care unit with acute exacerbations of COPD [12], and compare unfavorably for cancer patients 'the 5-year relative survival rate for persons diagnosed with cancer is 62.7%, with variation by cancer site and stage at diagnosis' [13]. This article examines why patients with severe advanced stage COPD do not receive appropriate palliative care simultaneously when receiving active treatment despite presenting with low-self-esteem, poor health status, low levels of physical activity, severe dyspnoea and fatigue compared with cancer patients. These symptoms are often associated with adverse events such as worsened functional impairment, frequent hospitalization, and premature death.

## Palliative care

A typical palliative care team may comprise of a physician, mental health and palliative care nurses, auxiliary staff, a pharmacist, bereavement counselor, psychologist, chaplain, social worker and volunteers etc. However because of cost implication and different settings of the palliative care services, the team may comprise some or all members of the team. The purpose is to maximize care, relieve suffering and improve quality of life for the patient and provide support for the family and carers with a team approach. The level of care may vary depending on the availability of staff and the set up of the palliative care programme, for example, inpatient or nursing home. In addition it may rely on the outcome of assessment of the palliative care team which may range from once daily or more to two or three times per week by the appropriate team members and with weekly review by the whole team.

Currently palliative care services are not widely available because of the high demand for care for patients with cancer and chronic progressive diseases which are costly to manage. However, a recent survey data in the US hospitals indicate an encouraging sign that the number of palliative care programs increased from 2000 [n = 632 (15%) of hospitals] to 2003 [n = 1027 (25%) of hospitals] [14].

Palliative care is not synonymous with terminal care and it should be apparent that palliative care approach focuses on symptom management, maintenance of a reasonable quality of life, good communication (patients, family members and physicians), increasing physical activities to maintain independence and practical support of emotional, spiritual and psychosocial support for patients and caregivers [15]. Currently, palliative care is mostly available for cancer patients who benefited from medical care to control physical symptoms of pain, dyspnoea, and emotional and spiritual support, at the end of life care [15]. However, this kind of provision is not widely available for COPD patients. Gore and co-workers [16] investigated the morbidity of end stage COPD in comparison to patients with unresectable non-small lung cancer (NSCLC) [FEV_1 _< 0.75 litres versus 1.47 litres]. The COPD patients were identified with poor quality of life, severe dyspnoea, and psychological burden of clinically relevant anxiety or depression (90% COPD versus 52% NSCLC). Despite having worse prognosis, the COPD patients received no palliative care compared with NSCLC patients. A recent systematic review [17] that investigated the prevalence of physical symptoms of five chronic progressive diseases (advanced cancer, AIDS, heart disease, renal disease, and COPD) identified common physical symptoms across the five conditions which were pain, breathlessness, and fatigue. In addition, a high prevalence of depression was reported in COPD patients. This indicates that palliative care is relevant for patients with all five chronic conditions. However the holistic aspects of care provision and management may require modification to satisfy individual patient's needs in order to improve quality of life and to support carers and families.

The impact of depression can be profound in influencing patient preferences of choice whether to receive a life sustaining treatment or not in patients with COPD [18]. In an elegant qualitative study, Curtis and colleagues [19] examined the differences in end-of-life care provision from patient perspectives for patients with COPD, Cancer and AIDS. The three groups differed in socio-demographic characteristics, and COPD patients were older compared with the Cancer and AIDS patients. However they have reported similar concern of whether the family physicians are skilled enough in providing end of life care (emotional support, communication and accessibility and continuity of care). The COPD patients further reported the desire for education to know more about the disease process, treatment, and prognosis, what dying might be like and advance care planning which may be a territory for the palliative care team to address.

## Why COPD patients do not receive palliative care?

General practitioners and healthcare professionals are challenged by prognostic accuracy of patient survival in patients with severe end-stage of COPD, and they are less likely to engage in end-of-life care planning in contrast with terminal diseases like cancer. This poses a challenge about when to refer patients with advanced stage of COPD for palliative care even though their physical, emotional and psychosocial needs are as severe as or worse than patients with lung cancer [16,20]. Furthermore there is a lack of resources which constraints for the wider availability of the palliative care programmes in the health care system.

Currently there is no research data to persuade health care providers that palliative care provision for advanced stage COPD patients has beneficial effects in terms of reducing the healthcare utilization (for example, hospital readmission) or improving quality of life in this patient group. However, a recent study [21] that investigated in male patients the benefits of palliative care provision (predominantly cancer including cardiovascular, pulmonary and gastrointestinal etc.) compared with the usual care (optimum medical treatment and hospital care provision). Patients that received palliative care were less likely to be admitted to intensive care unit during hospitalization, incur lower inpatient costs per day, and receive better medical care provision compared to the usual care patients. Hence palliative care may help in reducing health care costs and may avoid admission into the intensive care unit for patients with COPD.

Acute exacerbations are common in patients with severe COPD which require on average three-to-four times hospital readmission per year and patients are most likely to present with low self-esteem and suffer from a high level of depressive and anxiety symptoms. Our research group has reported recently that patients with severe acute exacerbation COPD identified at discharge with a co-morbid depression have increased likely hood of (13% more) of dying in the following year compared with non-depressed patients [11]. This observation indicates the lack of holistic palliative care provision for psychological, social support (patient and carers) and spiritual care at the end stage of COPD.

Patients with severe COPD are most likely to be housebound, socially isolated, physically disabled, and lead poor quality of life. They can be a source of concern for family and carers, as some patients are chair-bound, and may be in need of continuous support and care including routine basic daily activities for example, bathing.

## Benefits of palliative care for COPD patients

Palliative care for COPD patients will open an opportunity for better communication with patient, family, and physician in order to plan appropriate treatment strategies including advance care planning, and patient preferences with regard to choice of end of life withholding or withdrawing medical treatment and hospice care. For the details of topics and protocol guidelines to enhance patient-doctor-communication and treatment options see reviews [15,33].

The multidisciplinary nature of palliative care provision may encourage COPD patients to play an active role in self-management in order to optimize energy and function. In addition, the psychosocial support and the health care professionals' care and attention may promote confidence to over-come the disproportionate fear dyspnoea on exertion brings may help to engage in routine daily activities.

It will also provide unmet needs of the patients psychological, spiritual, and psychosocial needs in the advance stage of the disease in order to improve patients' quality of life. In addition, the spouse and/or carers are more likely to suffer from anxiety and depressive symptoms providing continuous care during the advance stage of the disease and end of life care without having any periods of respite. Therefore, it will open access to a specialist palliative care team including home nursing services and referral to hospice care.

## What are the potential barriers?

Table 1 summarizes some of the potential barriers [22-24] from the patients and the general practitioners perspectives to provide satisfactory care for the patients at the end stage of the disease. It requires a multi-modal approach to tackle the barriers as follows.

**Table 1:** Potential barriers of discussing the prognosis end-stage of COPD [22-24]

**Patients**	**General practitioners**
1) Unwillingness to discuss end of life care 2) Lack of communication (not sure which doctor will be taking care of me) 3) Ignoring not to discuss the issue 4) Lack of knowledge what type of care available 5) Loss of hope	1) Lack of confidence (ill-prepared to discuss the issue adequately) 2) Lack of time in busy surgery (increasing workload) 3) Uncertain about the information to provide about the prognosis in advance COPD 4) Lack of patient education about the end stage of COPD 5) Not in the priority list 6) Lack of resources and facilities

The general practitioners may require further training on communication skills in how to break sensitive information such as the prognosis of the disease and advance care planning [24]. It is also possible that general practitioners may not have adequate time with increased workload to discuss such kind of issues at great-depth. Hence, referring those patients in advance stage of the disease to palliative care team is a worthy endeavor.

Education of the patients and carers about the disease, including the prognostic and advance care planning at the early stage of the disease should be part of the pulmonary rehabilitation program. This may increase patient's awareness and confidence to ask relevant questions at the end-stage of the disease and to have a meaningful conversation with their physicians in order to devise appropriate treatment strategies. In order to achieve these goals further training is required to specialize on palliative care for the pulmonary rehabilitation team and others (for example, advanced practice nurses) that may be involved in patient care to assist the general practitioners.

## Assessment

Accurate assessment of the patients' psychological morbidity, physical disability and impaired quality of life, is the first step of towards planning good end of life care. A recent study in our department identified physical disability, poor quality of life and depression as predictors of mortality following the events of acute exacerbations of COPD in a preceding year [11]. The Manchester Respiratory Activities of Daily Living (MRADL) [25] is a self-administered physical disability scale, and is specifically validated as a postal questionnaire for patients with COPD. The score ranges from 0 to 21, and a low score corresponds to severe physical disability in activities of daily living. It takes less than five minutes to complete. The MRADL [11] score < 10 showed predictive values for one year mortality (sensitivity 75% and specificity 63%), i.e. good at identifying mortality but less good at predicting survival. The MRADL has a potential in identifying (selecting) severe COPD patients with physical disability that are most likely to benefit from palliative care provision while pursuing optimum medical treatment. The MRADL requires testing in future research. Others have suggested the BODE index (body mass index, airflow obstruction, dyspnoea and exercise capacity) [26,27] might be a useful tool as changes may relate to progression of disease and predicts mortality in this patient group.

## Treatment that may benefit COPD patients during palliative care provision

### Long acting bronchodilators

It is important to be aware of the effects of drugs to keep the airways open for a long period time in order to reduce breathlessness and increase quality of sleep during the night for the patient suffering from severe dyspnoea. Tiotropium (tiotropium bromide) is a safe long acting inhaled bronchodilator drug with a long duration of providing sustained bronchodilation throughout the day, and is relatively selective for muscarine M_1 _and M_3 _receptors, dissociating more quickly from M_2 _receptors [28,29]. A recent meta-analysis showed that Tiotropium reduced frequency of COPD exacerbations, relieved dyspnoea, and improved quality of life in patients with COPD [30]. Once daily dosing is convenient for patients but close monitoring for adverse effects such as dry mouth and urinary tract infections are essential.

### Oxygen Therapy

In the advanced stage of the disease, COPD patients often experience chronic hypoxia which may require oxygen therapy as it helps to relieve dyspnoea and improve physical activities within a home environment. However, the long-term benefits of oxygen therapy for COPD patients remain inconclusive. A few studies have reported that patients with COPD on long-term oxygen therapy (LTOT) suffer from high level of anxiety and depressive symptoms, poor quality of life [31] and premature death compared to non-LTOT patients with COPD [11].

### Opiates

Morphine is commonly used for patients with intolerable dyspnoea in palliative medicine. It helps the patient to feel comfortable, reduce breathlessness and improves the quality of sleeping pattern. Dosing depends on the symptom burden and the patient history of exposure. A recent review [32] supports the use of opioids to treat dyspnoea (reducing the sensation of breathlessness) in patients with advanced progressive diseases, including COPD. However, close monitoring of the patient condition for side effects such as nausea, vomiting, dizziness and constipation is essential. Morison and Morison [33] advise when ever possible to use the lowest effective dose of opioid medication and titrate the bowel regimen accordingly.

### Benzodiazepines

They can be considered in the treatment of severe dyspnoea and anxiety symptoms including panic attack which are common in patients with advance stage of COPD for example, regular low-dose longer-acting benzodiazepines such as diazepam 2–5 mg every 8 hours [34].

### Psychosocial and spiritual needs

COPD patients with severe dyspnoea, tiredness and pain may compromise their physical, psychological, social, and spiritual aspects of their lives. Palliative care (a multidisciplinary care) holistic care approach addressing some of the issues of may be a benefit for patients and families. However, to-date the benefits of spiritual care has not been explored in patients with COPD. It was reported that spiritual and religious beliefs can play an important role at the end of life care, for example, a question "are you at peace?" offers a patient an opportunity to express his/her spiritual concerns and to have a dialogue with the doctor 'in treatment decisions for patients and families, particularly with regard to initiation of and continuation of life-prolonging therapies' [35]. In this regard chaplains and local church minister may play an active role to support the patient and family in spiritual care.

### Pulmonary rehabilitation

There is strong evidence to suggest that a group based pulmonary rehabilitation program improves quality of life, exercise capacity, and increases confidence to pursue enjoyable hobbies for mild to moderate COPD patients. However, patients with the advanced stage COPD with physical and psychological symptoms (Table 2) especially those housebound may derive-benefits, if the exercise program is individually tailored, instructed and periodically supervised by the therapist as a part of home exercise program in that it may improve independence in physical activities and self-management. It is worth exploring whether the palliative care programme has any additional benefits when simultaneously provided with pulmonary rehabilitation programme.

**Table 2:** Indicators of physical symptoms with advanced end stage of COPD

Social isolation
Depression
Anxiety
Poor quality of life
Intolerable dyspnoea
Frequent hospital admissions
Housebound or chair bound
Fatigue (excessive tiredness)
Loss of hobbies
Loss of weight
Low self-esteem
Long term oxygen therapy
FEV_1 _< 30%

FEV_1 _= Percentage of forced expiratory volume in one second.

Patients with severe dyspnoea may benefit from relaxation therapy and breathing training (breathing control) exercises, appropriate positioning and advice on postural correction can also be useful in reducing sensation of breathlessness. Furthermore, providing a supportive listening environment, psychosocial counseling and reassuring patients and respite care for caregivers may also be beneficial. Devising coping strategies, adaptation of the home environment, energy conservation techniques to improve daily activities, for example, the inability to take a shower while standing upright can be overcome by placing a chair in the bath/shower-room are worthy of consideration. Malnutrition is common in advanced stage COPD. Factors that contribute to this are multifactiorial and may include the increased work of breathing because of severity of lung impairment, decreased appetite and a lack of balanced diet. The dietitian may provide an advice to the patient and carers about diet and supplementary diet intake. Patients with severe dyspnoea while eating may benefit from dividing the daily intake into several small meals.

In summary, palliative care is not available for patients with advanced stage COPD despite their having a poor prognosis with lower levels of self-efficacy, greater disability, poor quality of life, and higher levels of anxiety and depression worse than subjects with terminal non-small cell lung cancer. COPD patients are twice as likely to be admitted to an intensive care unit compared with lung cancer patients. Indeed, COPD is a chronic incurable disease, especially those in advanced stage of the disease pursuing intensive medical treatment may benefit from the simultaneous provision of the holistic care approach of palliative care services, medical services and social services to improve quality of end-of-life care.
